# Synergistic Effects of Riluzole and Sodium Butyrate on Barrier Function and Disease Progression of Amyotrophic Lateral Sclerosis Through the Gut‐Neuron Axis

**DOI:** 10.1002/cph4.70009

**Published:** 2025-04-02

**Authors:** Yongguo Zhang, KaReisha Robinson, Yinglin Xia, Jun Sun

**Affiliations:** ^1^ Division of Gastroenterology and Hepatology, Department of Medicine University of Illinois Chicago Chicago Illinois USA; ^2^ Jesse Brown VA Medical Center Chicago Illinois USA

**Keywords:** Amyotrophic lateral sclerosis, barrier function, BBB, Claudin‐5, gut‐brain axis, inflammation, Neuron, tight junctions, ZO‐1

## Abstract

Emerging evidence has shown that gut‐brain barrier dysfunction occurs at the early stages of ALS. Previous studies demonstrated that sodium butyrate significantly prolonged the life span of ALS mice. Riluzole is the first FDA‐approved drug for ALS treatment. We hypothesize that Riluzole and sodium butyrate combined treatment further decreases aggregation of the h‐SOD1^G93A^, restores the gut‐brain barrier function, and delays ALS progression. SOD1^G93A^ mice (9–10‐week‐old) were treated with Riluzole (10 mg/kg, I.P. daily), sodium butyrate (2% in drinking water), or Riluzole and sodium butyrate combination for 6 weeks. The Riluzole/butyrate combination showed a significantly longer rotarod time, increased grip strength, and enhanced intestinal barrier, as compared with Riluzole or sodium butyrate‐only treatment. More reduction of h‐SOD1^G93A^ aggregation was observed in the colon, spinal cord lumbar, and brain cortex with Riluzole and sodium butyrate combination, compared with Riluzole or sodium butyrate‐only treatment. Tight junction proteins (ZO‐1 and Claudin‐5) significantly increased in the colon, spinal cord lumbar, and brain cortex of mice with Riluzole and sodium butyrate treatment. The Riluzole and sodium butyrate combination reduced serum lipopolysaccharides and h‐SOD1^G93A^ aggregation, and inflammatory cytokines more than those in Riluzole or sodium butyrate‐only treatment. Overall, Riluzole and sodium butyrate treatment is more effective than either Riluzole or sodium butyrate‐only in delaying ALS progress. It provides a potential therapeutic strategy and mechanism by restoring barrier function through the gut‐brain axis for ALS.

## Graphical abstract

Gut‐brain barrier dysfunction occurs in amyotrophic lateral sclerosis (ALS) along with other symptoms such as muscle weakness, muscle twitching, and difficulty with speech. Combined treatment with FDA‐approved Riluzole and sodium butyrate is more efficient than either Riluzole or sodium butyrate alone in delaying ALS progress, likely through the gut–neuron axis.

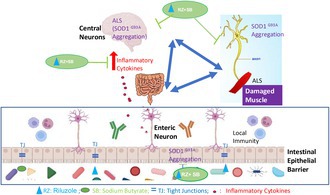

## 1 Introduction

Amyotrophic lateral sclerosis (ALS) is a fatal neuromuscular disease characterized by progressive motor neuron death and skeletal muscle atrophy and paralysis (Duranti and Villa 2023; Hardiman et al. 2017). The lifetime risk of ALS is about 1 in 472 in women and 1 in 350 in men (Alonso et al. 2009). Because ALS is an age‐dependent disease, an increase in the prevalence of ALS can be anticipated as the US population increases and ages. Military veterans, regardless of the branch of service, the era in which they served, or whether they served during a time of peace or war, are at a greater risk of dying from ALS than those who have not served in the military (Beard et al. 2016). Many cases of familial ALS (20%–25% of familial ALS cases) are associated with mutations in the Cu/Zn superoxide dismutase gene (SOD1). The SOD1 gene protects cells from oxygen molecules. Yet, when SOD1 is mutated, oxygen molecules have access to cells, which induces glutamate, thereby leading to neuronal injury. Most patients with ALS die within 5 years after the disease onset. Despite intensive research efforts, there is no effective cure for ALS. The available treatments (Riluzole and Edaravone) are to relieve symptoms and improve the quality of life for patients with ALS. Qalsody was most recently approved by the US Food and Drug Administration and targets the SOD1 mRNA to prevent the production of the SOD1 protein. There are still significant needs to develop novel treatments for ALS and improve the quality of life of ALS patients (Sun and Zhang 2024).

Riluzole was the first FDA‐approved treatment for ALS and is speculated to reduce glutamatergic neurotransmission by blocking voltage‐gated sodium channels on presynaptic neurons. Riluzole extends the patient's life span for a few months (Joyce et al. 2011). Increased serum inflammatory cytokines (Zhang et al. 2009), bacterial lipopolysaccharides (LPS) (Zhang et al. 2009), and aberrant microbiome have been reported in prior studies of human ALS (Fang et al. 2016). Our study with oral administration of sodium butyrate presents a promising therapeutic strategy (Sun and Zhang 2024; Zhang et al. 2017, 2021). Mice with butyrate treatment showed a significantly longer latency to fall in the rotarod test, compared with the G93A non‐treatment mice. Bacterial LPS, glycolipids found in the outer membrane of gram‐negative bacteria, are pro‐inflammatory and have been found to be increased in the serum of ALS patients (Zhang et al. 2009). Indeed, increased inflammatory cytokines IL‐17 and IL‐23 have been reported in the serum and cerebrospinal fluid of patients with ALS (Rentzos et al. 2010). We have demonstrated increased serum IL‐17 in the SOD1^G93A^ ALS mice, compared to the wild‐type mice (Wu et al. 2015). Our study showed that dysbiosis was correlated with intestinal inflammation and increased intestinal permeability in ALS (Wu et al. 2015; Figueroa‐Romero et al. 2019). However, the impact and mechanism of Riluzole in the ALS intestine are unknown. The combined role and mechanism of Riluzole and sodium butyrate are also unknown.

In the current study, we use the 2% sodium butyrate in the drinking water combined with the Riluzole in the SOD1^G93A^ mice. The outcomes focused on clinical disease progression, e.g., body weight, ratorad, onset, and neuroprotection (reducing motor neuron loss and inhibiting glial activation, and survival). Moreover, we examined the changes in aggregation of human mutant SOD1^G93A^ (h‐SOD1^G93A^), tight junctions (TJs) for barrier function, and inflammation. A better understanding of the mechanisms of existing drugs and the synergistic effects of Riluzole and sodium butyrate will provide insights into barrier function and ALS progression.

## 2 Materials and Methods

### 2.1 Animal Model

SOD1^G93A^ strain (B6SJL‐Tg (SOD1‐G93A) 1Gur/J, stock No. 002726) (Zhang et al. 2017, 2021; Ogbu et al. 2022; Sun 2017) was purchased from Jackson Laboratory (The Jackson Laboratory, Bar Harbor, ME, USA). All mice were housed in specific pathogen‐free environments under a controlled condition of 12 h light/12 h dark cycle at 20°C–22°C and 45% ± 5% humidity, with free access to the same food and autoclaved water. All materials involved, including cage, bedding, water bottles, and cage card holder, were autoclaved before housing mice. The mice are housed of the same gender, and each cage has no more than 5 mice. All experiments were carried out in strict accordance with the recommendations in the Guide for the Care and Use of Laboratory Animals of the National Institutes of Health. The protocol was approved by the IACUC of the University of Illinois Chicago Committee on Animal Resources (ACC 21‐178 and ACC 23‐149). The study is reported in accordance with ARRIVE guidelines.

### 2.2 Rotarod Test

Motor coordination, endurance, and balance were determined in latency by a rotarod test. Mice were trained on a rotarod test 3 days before performance trials at an accelerating speed from 4 to 40 rpm for 300 s using the Rotarod Model LE8205 (Harvard Apparatus, Holliston, MA, USA). Latency to fall was recorded when the mouse fell from the rod (Zhang et al. 2021; Gilli et al. 2016). Each mouse was tested in 2 trials per day for 2 consecutive days. The mean times for 4 trials of the tests were calculated for each mouse.

### 2.3 Assessment of Grip Strength

Forelimb and hindlimb grip measurements were acquired in triplicate with a 25 N Grip strength meter (Harvard Apparatus, Holliston, MA, USA). The mice were lowered onto a triangle bar of the grip strength meter until the animals gripped the bar with their forelimbs or hindlimbs; then the mice were pulled gently backward until they released their grip. The force gauge of the grip meter recorded the maximum force (Zhang et al. 2021; Huerta Ojeda et al. 2021).

### 2.4 Riluzole and Butyrate Treatment in SOD1^G93A^ Mice

SOD1^G93A^ mice (male and female) aged 9–10 weeks were randomly assigned to 4 groups. The control group received no treatment. The treatment of 2% sodium butyrate in filtered drinking water and the dose of Riluzole (10 mg/kg, intraperitoneally) were based on previous studies (Verhave et al. 2012; Kitzman 2009; Milane et al. 2009), including ours (Zhang et al. 2017, 2021). The treatment with 2% sodium butyrate in filtered drinking water led to restored intestinal microbial homeostasis, reduced human‐SOD1^G93A^ aggregation, enhanced ENS and muscle function in intestine and neurons, and prolonged life span of SOD1^G93A^ mice (Zhang et al. 2017, 2021). The dose of Riluzole (10 mg/kg, intraperitoneally) was beneficial in neurodegenerative disease (Verhave et al. 2012; Kitzman 2009; Milane et al. 2009). The Riluzole‐treated group intraperitoneally received Riluzole (Sigma‐Aldrich, 1604337, St. Louis, MO, USA) at a dose of 10 mg/kg body weight daily. The sodium butyrate‐treated group received 2% sodium butyrate (Sigma‐Aldrich, 303410, St. Louis, MO, USA) in filtered drinking water. The Riluzole/sodium butyrate combination‐treated group received Riluzole and sodium butyrate. The Riluzole and sodium butyrate treatments started at 9–10 weeks and finished at 15–16 weeks. All animals were weighed and received a detailed clinical examination, which included assessments of appearance, movement and behavior patterns, skin and hair conditions, eyes and mucous membranes, respiration, and excreta. Restricted outstretching of the hind legs observed on tail holding was considered a symptom of ALS; mice that were unable to turn over within 20 s after being laid on their backs were humanely euthanized by CO_2_ inhalation followed by cervical dislocation.

### 2.5 Western Blot Analysis and Antibodies

Mice colonic epithelial cells were collected by scraping the tissue from the colon of the mouse, including the proximal and distal regions. Mice spinal cord lumbar and brain cortex were harvested and minced into tiny pieces by using scissors (Shen et al. 2023; Lin et al. 2016). The collected tissues were sonicated in lysis buffer (10 mM Tris, pH 7.4, 150 mM NaCl, 1 mM EDTA, 1 mM EGTA, pH 8.0, 1% Triton X‐100) with 0.2 mM sodium ortho‐vanadate and protease inhibitor cocktail. The protein concentration was measured using the BioRad Reagent (BioRad, Hercules, CA, USA) and then sonicated. Equal amounts of protein were separated by SDS‐polyacrylamide gel electrophoresis, transferred to nitrocellulose, and immunoblotted with primary antibodies. The following antibodies were used: anti‐human SOD1 (Abcam, ab52950, Cambridge, MA, USA), anti‐ZO‐1 (Invitrogen, 33‐9100, Carlsbad, CA, USA), anti‐Claudin‐5 (Invitrogen, 35‐2500, Carlsbad, CA, USA), anti‐Claudin‐1 (Invitrogen, 71‐7800, Carlsbad, CA, USA), anti‐GAPDH (Cell signaling technology, 5174, Danvers, MA, USA), or anti‐β‐actin (Sigma‐Aldrich, A5316, St. Louis, MO, USA) antibodies and were visualized by ECL (Thermo Fisher Scientific, Waltham, MA, USA). Membranes that were probed with more than one antibody were stripped before re‐probing. The software Quantity One has been used for the quantification of the western blot bands. Briefly, the “rectangular tool” was first selected to measure the background and the bands of western blots one by one. All the values of “density” and “volume” after measurement were transferred to an excel file. With the subtraction of background measurement, the “density” values for each band on the western blot were calculated.

### 2.6 Immunofluorescence

The colonic, spinal cord, and brain tissues were freshly isolated and embedded in paraffin wax after fixation with 10% neutral buffered formalin. Immunofluorescence was performed on paraffin‐embedded sections (5 μm). After preparation of the slides as described previously (Zhang et al. 2022; Zhang, Zhang, et al. 2023), tissue samples were incubated with anti‐human SOD1 (Abcam, ab52950, Cambridge, MA, USA), anti‐ZO‐1 (Invitrogen, 33‐9100, Carlsbad, CA, USA), and anti‐Claudin‐5 (Invitrogen, 35‐2500, Carlsbad, CA, USA) at 4°C overnight. Samples were then incubated with goat anti‐rabbit Alexa Flour 488 (Invitrogen, A‐11008, Carlsbad, CA, USA), goat anti‐mouse Alexa Flour 488 (Invitrogen, A‐11001, Carlsbad, CA, USA) or goat anti‐mouse Alexa Flour 594 (Invitrogen, A‐11032, Carlsbad, CA, USA) and DAPI (Invitrogen, D1306, Carlsbad, CA, USA) for 1 h at room temperature. Tissues were mounted with SlowFade (Invitrogen, s2828, Carlsbad, CA, USA), followed by a coverslip, and the edges were sealed to prevent drying. Specimens were examined with a Zeiss laser scanning microscope LSM 710 (Carl Zeiss Inc., Oberkochen, Germany). The h‐SOD1^G93A^ protein aggregation was quantified with AggreCount software (https://aggrecount.github.io/). Fluorescence intensity was determined by using Image J software. This method determines the corrected total fluorescence by subtracting out background signal, which is useful for comparing the fluorescence intensity between cells or regions.

### 2.7 Intestinal Permeability

Fluorescein isothiocyanate‐dextran (average molecular weight 4000, Sigma, 46944, Burbank, CA, USA, diluted in HBSS) was gavaged (25 mg/kg mouse) 4 h before sample harvest. Mice were anesthetized with avertin; depth of anesthesia was assessed with toe pinch, and then blood was collected via cardiac puncture followed by cervical dislocation. Mouse blood samples were collected for intestinal permeability test (Zhang et al. 2019, 2020; Zhang, Xia, et al. 2023).

### 2.8 Serum Lipopolysaccharides (LPS) Detection

LPS in serum samples was measured with limulus amebocyte lysate chromogenic end point assays (Hycult Biotech, HIT302, Plymouth, PA, USA) according to the manufacturer's indications. The samples were diluted 1:4 with endotoxin‐free water and then heated at 75°C for 5 min on a warm plate to denature the protein before the reaction. A standard curve was generated and used to calculate the concentrations, which were expressed as EU/mL in the serum samples.

### 2.9 Multiplex ELISA Assay

Mouse blood samples were collected by cardiac puncture and placed in tubes containing EDTA (10 mg/mL). Mouse cytokines were measured using a Cytokine and Chemokine Convenience 26‐Plex Mouse ProcartaPlex Panel 1 (Invitrogen, EPXR260‐26088‐901, Carlsbad, CA, USA) according to the manufacturer's instructions. Briefly, beads of defined spectral properties were conjugated to protein‐specific capture antibodies and added along with samples (including standards of known protein concentration, control samples, and test samples) into the wells of a filter‐bottom microplate, where proteins bound to the capture antibodies over the course of a 2‐h incubation. After washing the beads, protein‐specific biotinylated detector antibodies were added and incubated with the beads for 1 h. After removal of excess biotinylated detector antibodies, the streptavidin‐conjugated fluorescent protein R‐phycoerythrin was added and allowed to incubate for 30 min. After washing to remove unbound streptavidin–R‐phycoerythrin, the beads were analyzed with the Luminex detection system (Bio‐Rad, Bio‐Plex 200 Systems, Hercules, CA, USA).

### 2.10 Statistical Analysis

All data were expressed as the mean ± SEM or ± SD. All statistical tests were 2‐sided. All *p*‐values < 0.05 were considered statistically significant. The differences between samples for more than two groups were analyzed using one‐way ANOVA or two‐way ANOVA as appropriate based on data distribution and the number of factors, respectively. The *p*‐values in ANOVA analyses were adjusted for correction of multiple comparisons using the Tukey method to ensure accurate results. Statistical analyses were performed using GraphPad Prism 8 (GraphPad Inc., San Diego, CA, USA).

## 3 Results

### 3.1 Riluzole and Sodium Butyrate Combination Showed a Significantly Synergistic Protection Against Disease Progression in SOD1^G93A^ Mice

**FIGURE 1:**
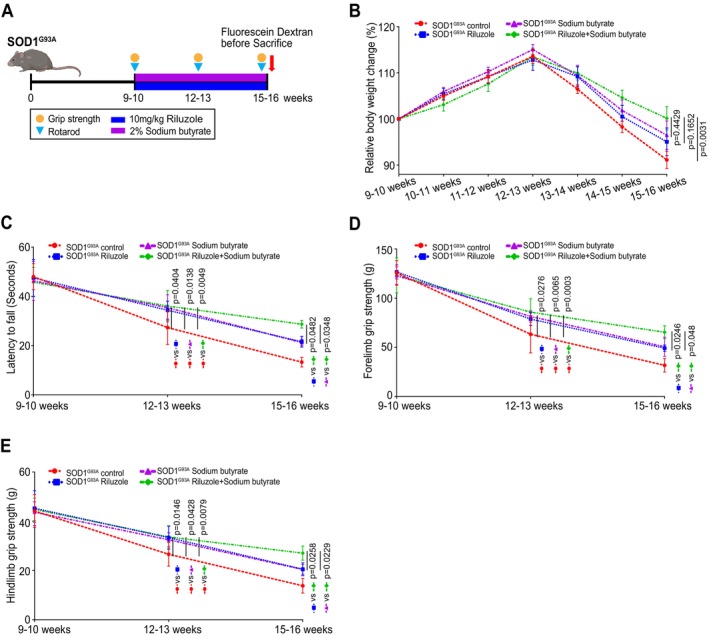
Riluzole and sodium butyrate combination showed a significantly delayed disease progress compared with Riluzole or sodium butyrate‐only treatment. (A) Schematic overview of the SOD1^G93A^ mice treated with Riluzole, sodium butyrate, or Riluzole and sodium butyrate combination in SOD1^G93A^ mice (9‐ to 10‐week‐old). Riluzole was I.P. at a dose of 10 mg/kg body weight daily. 2% sodium butyrate was given in filtered drinking water. The treatments lasted 6 weeks. (B) Body weight changes of the SOD1^G93A^ mice. At the age of 15–16 weeks, the Riluzole and sodium butyrate combination showed a significant less weight loss, compared to the no‐treatment SOD1^G93A^ mice. Data are expressed as mean ± SEM. *n* = 8–10, two‐way ANOVA test. (C) Riluzole and sodium butyrate combination‐treated SOD1^G93A^ mice had a significantly increased rotarod time compared to Riluzole‐only, sodium butyrate‐only‐treated SOD1^G93A^ mice. Data are expressed as mean ± SD. *n* = 8–10, two‐way ANOVA test. (D) Riluzole and sodium butyrate combination led to a significantly increased forelimb grip strength and (E) hindlimb grip strength, compared to Riluzole‐only, or sodium butyrate‐only‐treated SOD1^G93A^ mice. From the age of 12–13 weeks, Riluzole‐only, sodium butyrate‐only, or Riluzole and sodium butyrate combination‐treated SOD1^G93A^ mice all had a significantly increased forelimb grip strength and hindlimb grip strength compared to control mice. At the age of 15–16 weeks, Riluzole and sodium butyrate combination‐treated SOD1^G93A^ mice had a significantly increased forelimb grip strength and hindlimb grip strength, compared to Riluzole‐only, or sodium butyrate‐only‐treated SOD1^G93A^ mice. Data are expressed as mean ± SD. *n* = 8–10, two‐way ANOVA test. All *p‐*values are shown in the figures.

To examine the synergistic effects of Riluzole and sodium butyrate on disease progression, we set up 4 experimental groups with Riluzole‐only, sodium butyrate‐only, and Riluzole and sodium butyrate combination, and control without treatment in SOD1^G93A^ mice aged 9–10 weeks. Mice were randomly assigned to each group. The treatment lasted a total of 6 weeks. The Riluzole‐treated group received Riluzole via I.P. at a dose of 10 mg/kg of body weight daily. The sodium butyrate‐treated group received 2% sodium butyrate in filtered drinking water (Figure 1A). The date of disease onset is defined as the first day when a mouse fails the 15‐rpm rotarod task and starts to show muscle tremors in the hind limbs (Ito et al. 2008; Xu et al. 2006). The body weight will be monitored every day during the treatment period. The peak body weight is a reasonable and consistent determinant of onset, particularly in combination with a measure of neurological score (Ludolph et al. 2007; Scott et al. 2008). Body weight changes are shown in Figure 1B. From the age of 13–14 weeks, Riluzole‐only, sodium butyrate‐only or Riluzole and sodium butyrate combination‐treated SOD1^G93A^ mice groups show slight weight loss without statistical significance, compared to the no‐treatment SOD1^G93A^ mice. From the age of 14–15 weeks, Riluzole/sodium butyrate combination treatment showed a slight weight loss without statistical significance, compared to the Riluzole‐only or sodium butyrate‐only treatment SOD1^G93A^ mice. At the age of 15–16 weeks, Riluzole and sodium butyrate combination showed significantly less weight loss compared to the no‐treatment SOD1^G93A^ mice.

The SOD1^G93A^ mice were tested on an accelerating rotarod (Xu et al. 2006) to examine the neuromuscular activity performance. The Riluzole and sodium butyrate combination‐treated SOD1^G93A^ mice had a significantly increased rotarod time, compared to Riluzole‐only and sodium butyrate‐only‐treated SOD1^G93A^ mice (Figure 1C). From the age of 12–13 weeks, Riluzole‐only, sodium butyrate‐only, or Riluzole and sodium butyrate combination‐treated SOD1^G93A^ mice all had a significantly increased rotarod time, compared to control mice without treatment. At the age of 15–16 weeks, the combination group had a significantly increased rotarod time, compared to SOD1^G93A^ mice with only Riluzole or sodium butyrate. Riluzole and sodium butyrate combination‐treated SOD1^G93A^ mice had a significantly increased strength of forelimb grip (Figure 1D) and hindlimb grip (Figure 1E), compared to Riluzole‐only or sodium butyrate‐only‐treated SOD1^G93A^ mice. From the age of 12–13 weeks, Riluzole‐only, sodium butyrate‐only, or Riluzole and sodium butyrate combination‐treated SOD1^G93A^ mice all had a significantly increased forelimb grip strength and hindlimb grip strength, compared to control mice without treatment. At the age of 15–16 weeks, Riluzole and sodium butyrate combination‐treated SOD1^G93A^ mice had a significantly increased forelimb grip strength and hindlimb grip strength, compared to Riluzole‐only or sodium butyrate‐only‐treated SOD1^G93A^ mice. The date of death is defined as the day when the mouse cannot right itself within 30 s after being placed on its back (Xu et al. 2006). We did not perform the survival curve of the SOD1^G93A^ mice with and without the treatment in ALS progression due to the restrictions of the animal protocol. Overall, our data suggested that Riluzole and sodium butyrate combination had a significantly protective effect against disease progression in SOD1^G93A^ mice.

### 3.2 Riluzole and Sodium Butyrate Combined Treatment Preserved the Permeability and Reduced h‐SOD1^G93A^ Aggregation in the SOD1^G93A^ Mice

**FIGURE 2:**
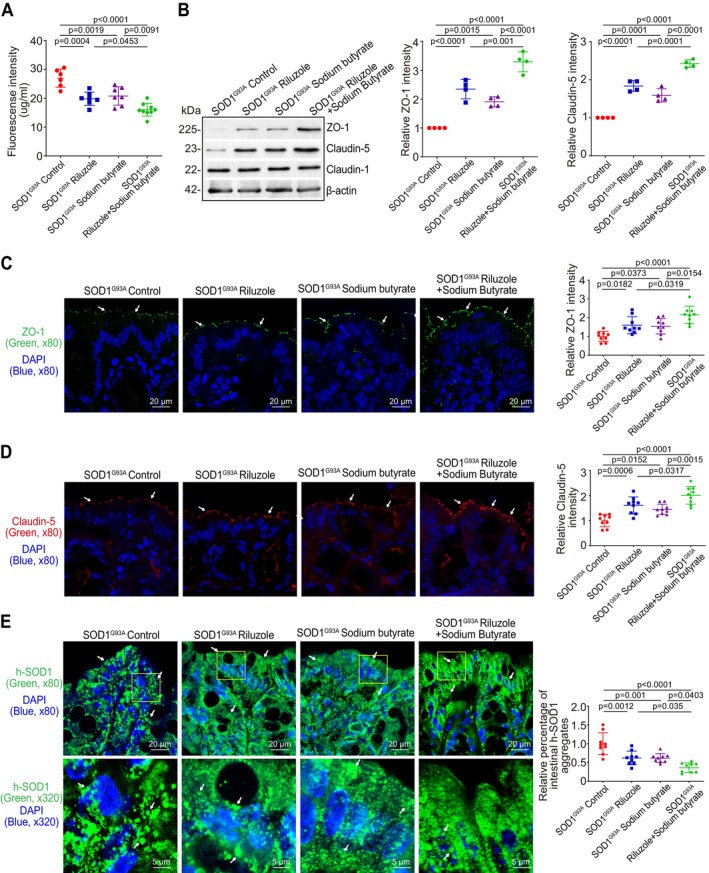
Decreased permeability, reduced h‐SOD1^G93A^ aggregation, and increased expression of tight junction proteins in the colon of SOD1^G93A^ mice with Riluzole/sodium butyrate combined treatment. (A) Intestinal permeability was decreased in all Riluzole‐only, sodium butyrate‐only, or Riluzole and sodium butyrate combination‐treated SOD1^G93A^ mice groups, compared to control mice. Meanwhile, Riluzole and sodium butyrate combination‐treated SOD1^G93A^ mice had a significantly decreased intestinal permeability, compared to Riluzole‐only or sodium butyrate‐only‐treated SOD1^G93A^ mice. Data are expressed as mean ± SD. *n* = 6–9, one‐way ANOVA test. (B) The expression of tight junction proteins, ZO‐1 and Claudin‐5, was increased in the colon of all Riluzole‐only, sodium butyrate‐only, or Riluzole and sodium butyrate combination‐treated SOD1^G93A^ mice groups compared to control mice. Meanwhile, Riluzole and sodium butyrate combination‐treated SOD1^G93A^ mice had a significantly increased ZO‐1 and Claudin‐5 expression in the colon compared to Riluzole‐only or sodium butyrate‐only‐treated SOD1^G93A^ mice by WB. Data are expressed as mean ± SD. *n* = 4, one‐way ANOVA test. (C) ZO‐1 and (D) Claudin‐5 expressions increased in intestines of all of Riluzole‐only, sodium butyrate‐only or Riluzole and sodium butyrate combination‐treated SOD1^G93A^ mice groups compared to control mice, as determined by IF staining. The relative fluorescence intensity was quantified with ImageJ by counting 3 images for each sample. Data are shown as mean ± SD, *n* = 3 per group, one‐way ANOVA test. (E) Decreased Aggregation of h‐SOD1^G93A^ in the colon of SOD1^G93A^ mice with Riluzole, sodium butyrate‐only, or combination, compared to control mice without treatment. Meanwhile, Riluzole and sodium butyrate combination‐treated SOD1^G93A^ mice had a significantly reduction of aggregated h‐SOD1^G93A^, compared to Riluzole‐only or sodium butyrate‐only‐treated SOD1^G93A^ mice. The h‐SOD1^G93A^ protein aggregation was quantified with AggreCount by counting 3 images for each sample. Data are shown as mean ± SD, *n* = 3 per group, one‐way ANOVA test. All *p*‐values are shown in the figures.

Increased gut permeability was reported in the SOD1^G93A^ mice (Zhang et al. 2017, 2021; Martin et al. 2022). We then examined whether treatment delays the intestinal dysfunction during ALS progression. Intestinal permeability was decreased in all Riluzole, sodium butyrate, or Riluzole and sodium butyrate combination‐treated SOD1^G93A^ mice groups, compared to control mice. Meanwhile, Riluzole and sodium butyrate combination‐treated SOD1^G93A^ mice had a significantly decreased intestinal permeability, compared to Riluzole‐only or sodium butyrate‐only‐treated SOD1^G93A^ mice (Figure 2A). The intestinal permeability was regulated by the TJs. We then examined the expression of TJ proteins. ZO‐1 and Claudin‐5 detected by Western Blot (WB) were increased in intestines of Riluzole, sodium butyrate, or Riluzole and sodium butyrate combination, compared to control mice without treatment. Meanwhile, Riluzole and sodium butyrate combination‐treated SOD1^G93A^ mice had the most significant increase in ZO‐1 and Claudin‐5 expression in the colon, compared to Riluzole‐only or sodium butyrate‐only‐treated SOD1^G93A^ mice (Figure 2B). ZO‐1 and Claudin‐5 expressions increased in the colon of all Riluzole‐only, sodium butyrate‐only, or Riluzole/sodium butyrate combination‐treated SOD1^G93A^ mice groups compared to control mice. Meanwhile, Riluzole and sodium butyrate combination‐treated SOD1^G93A^ mice had a significantly increased ZO‐1 and Claudin‐5 expression in the colon, compared to Riluzole‐only or sodium butyrate‐only‐treated SOD1^G93A^ mice, as determined by immunofluorescence (IF) staining (Figure 2C,D). The aggregation of the h‐SOD1 mutated protein was tested as an indicator of ALS progression. We also observed decreased aggregation of h‐SOD1^G93A^ in the colon of Riluzole, sodium butyrate, or Riluzole/sodium butyrate combination groups, compared to control mice without treatment. As expected, Riluzole and sodium butyrate combination‐treated SOD1^G93A^ mice had the most significant reduction in aggregation of intestinal h‐SOD1^G93A^, compared to Riluzole‐only or sodium butyrate‐only‐treated SOD1^G93A^ mice (Figure 2E).

### 3.3 In the Spinal Cord Lumbar, Riluzole and Sodium Butyrate Combined Treatment Reduced h‐SOD1^G93A^ Aggregation and Increased Expression of TJ Proteins in SOD1^G93A^ Mice

**FIGURE 3:**
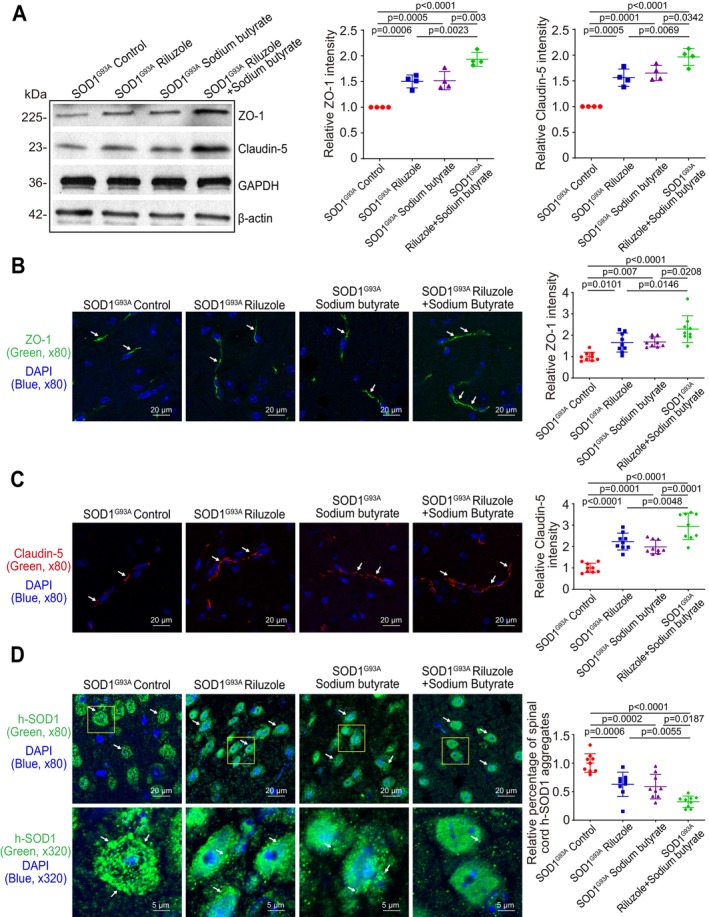
Reduced h‐SOD1^G93A^ aggregation and increased expression of ZO‐1 and Claudin‐5 in the spinal cord lumbar of Riluzole/sodium butyrate‐treated SOD1^G93A^ mice. (A) The expressions of ZO‐1 and Claudin‐5 were increased in the spinal cord of all Riluzole‐only, sodium butyrate‐only, or Riluzole and sodium butyrate combination groups‐treated SOD1^G93A^ mice, compared to control mice. Meanwhile, Riluzole and sodium butyrate combination‐treated SOD1^G93A^ mice had a significantly increased ZO‐1 and Claudin‐5 expression in the spinal cord lumbar, compared to Riluzole‐only or sodium butyrate‐only‐treated SOD1^G93A^ mice by WB. Data are expressed as mean ± SD. *n* = 4, one‐way ANOVA test. (B) ZO‐1 and (C) Claudin‐5 expressions increased in the spinal cord lumbar of all Riluzole‐only, sodium butyrate‐only, or Riluzole and sodium butyrate combination‐treated SOD1^G93A^ mice compared to control mice. Meanwhile, Riluzole and sodium butyrate combination‐treated SOD1^G93A^ mice had a significantly increased ZO‐1 and Claudin‐5 expression in the spinal cord lumbar, compared to Riluzole‐only and sodium butyrate‐only‐treated SOD1^G93A^ mice, as determined by IF staining. The relative fluorescence intensity was quantified with ImageJ by counting 3 images for each sample. Data are shown as mean ± SD, *n* = 3 per group, one‐way ANOVA test. (D) Aggregated h‐SOD1^G93A^ was decreased in the spinal cord lumbar of Riluzole, sodium butyrate, or Riluzole and sodium butyrate combination groups, compared to control mice. The h‐SOD1^G93A^ protein aggregation was quantified with AggreCount by counting 3 images for each sample. Data are shown as mean ± SD, *n* = 3 per group, one‐way ANOVA test. All *p*‐values are shown in the figures.

We further examined the TJ proteins in the spinal cord. As shown in Figure 3A, the expression of TJ proteins, ZO‐1 and Claudin‐5, was increased in the spinal cord lumbar of all Riluzole, sodium butyrate, or Riluzole and sodium butyrate combination groups, compared to control SOD1^G93A^ mice. Meanwhile, the Riluzole and sodium butyrate combination had the most significant increase in ZO‐1 and Claudin‐5 expression by WB. The distribution and density of ZO‐1 (Figure 3B) and Claudin‐5 (Figure 3C) had the most increase in the spinal cord lumbar of Riluzole and sodium butyrate‐treated SOD1^G93A^ mice, as determined by IF staining. Aggregated h‐SOD1^G93A^ decreased in the spinal cord lumbar of all Riluzole, sodium butyrate, or combination groups, compared to control mice (Figure 3D). Meanwhile, the Riluzole and sodium butyrate combination‐treated SOD1^G93A^ mice had the most significant decrease in aggregated h‐SOD1^G93A^ in the spinal cord lumbar, compared to Riluzole or sodium butyrate‐only‐treated SOD1^G93A^ mice.

### 3.4 Riluzole and Sodium Butyrate Combination Treated SOD1^G93A^ Mice Had Reduced h‐SOD1^G93A^ Aggregation and Increased ZO‐1 and Claudin‐5 in the Brain Cortex

**FIGURE 4:**
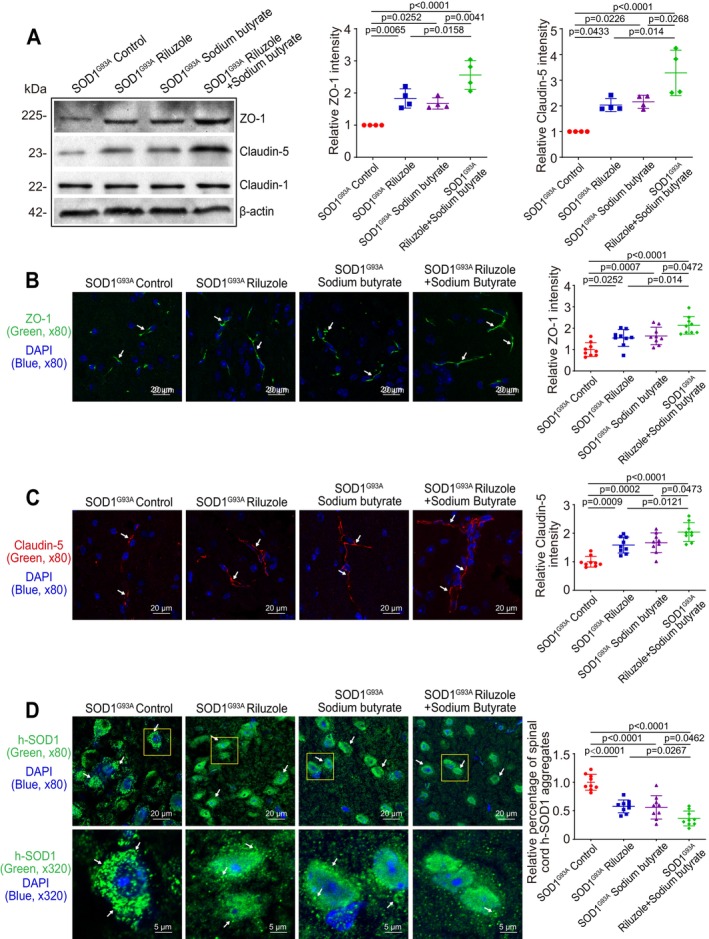
Reduced h‐SOD1^G93A^ aggregation and increased expression of ZO‐1 and Claudin‐5 in the brain cortex of Riluzole/sodium butyrate‐treated SOD1^G93A^ mice. (A) The expressions of ZO‐1 and Claudin‐5 were increased in the brain cortex of Riluzole‐only, sodium butyrate, or Riluzole and sodium butyrate combination‐treated groups, compared to control mice. Meanwhile, Riluzole and sodium butyrate combination‐treated SOD1^G93A^ mice had a significantly increased ZO‐1 and Claudin‐5 expression in the brain cortex, compared to Riluzole‐only or sodium butyrate‐only‐treated SOD1^G93A^ mice by WB. Data are expressed as mean ± SD. *n* = 4, one‐way ANOVA test. (B) ZO‐1 expression and (C) Claudin‐5 increased in the brain cortex of all Riluzole‐only, sodium butyrate‐only, or Riluzole and sodium butyrate combination groups‐treated SOD1^G93A^ mice, compared to control mice. Meanwhile, Riluzole and sodium butyrate combination‐treated SOD1^G93A^ mice had a significantly increased ZO‐1 and Claudin‐5 expression in the brain cortex, compared to Riluzole‐only or sodium butyrate‐only‐treated SOD1^G93A^ mice, as determined by IF staining. The relative fluorescence intensity was quantified with ImageJ by counting 3 images for each sample. Data are shown as mean ± SD, *n* = 3 per group, one‐way ANOVA test. (D) Aggregated h‐SOD1^G93A^ was decreased in the brain cortex of all Riluzole‐only, sodium butyrate‐only, or Riluzole and sodium butyrate combination‐treated SOD1^G93A^ mice groups, compared to control mice. Meanwhile, Riluzole and sodium butyrate combination‐treated SOD1^G93A^ mice had a significant decrease in the brain cortex of aggregation of h‐SOD1^G93A^, compared to Riluzole or sodium butyrate‐only‐treated SOD1^G93A^ mice. The h‐SOD1^G93A^ protein aggregation was quantified with AggreCount by counting 3 images for each sample. Data are shown as mean ± SD, *n* = 3 per group, one‐way ANOVA test. All *p*‐values are shown in the figures.

To test the hypothesis that Riluzole and sodium butyrate combined treatment better decreases aggregation of the h‐SOD1 mutated protein and restores the gut‐brain barrier function, thus delaying the ALS disease onset, we examined ZO‐1 and Claudin‐5 in the cortex of brain tissue. Riluzole and sodium butyrate combination had a significantly increased the expressions of ZO‐1 and Claudin‐5 in the brain cortex, compared to Riluzole or sodium butyrate‐only‐treated SOD1^G93A^ mice by WB (Figure 4A). As expected, Riluzole and sodium butyrate combination‐treated SOD1^G93A^ mice had a significantly increased ZO‐1 and Claudin‐5 expression in the brain cortex, compared to Riluzole‐only or sodium butyrate‐only‐treated SOD1^G93A^ mice, as determined by IF staining (Figure 4B,C). Claudin‐1 expression showed no change in the brain cortex. In the meanwhile, the h‐SOD1^G93A^ protein aggregation was quantified with AggreCount. Riluzole and sodium butyrate‐treated SOD1^G93A^ mice had a significant decrease in the brain cortex aggregation h‐SOD1^G93A^, compared to Riluzole‐only or sodium butyrate‐only‐treated SOD1^G93A^ mice (Figure 4D).

### 3.5 Riluzole and Sodium Butyrate Combined Treatment Reduced Serum LPS and Inflammatory Cytokines in SOD1^G93A^ Mice

**FIGURE 5:**
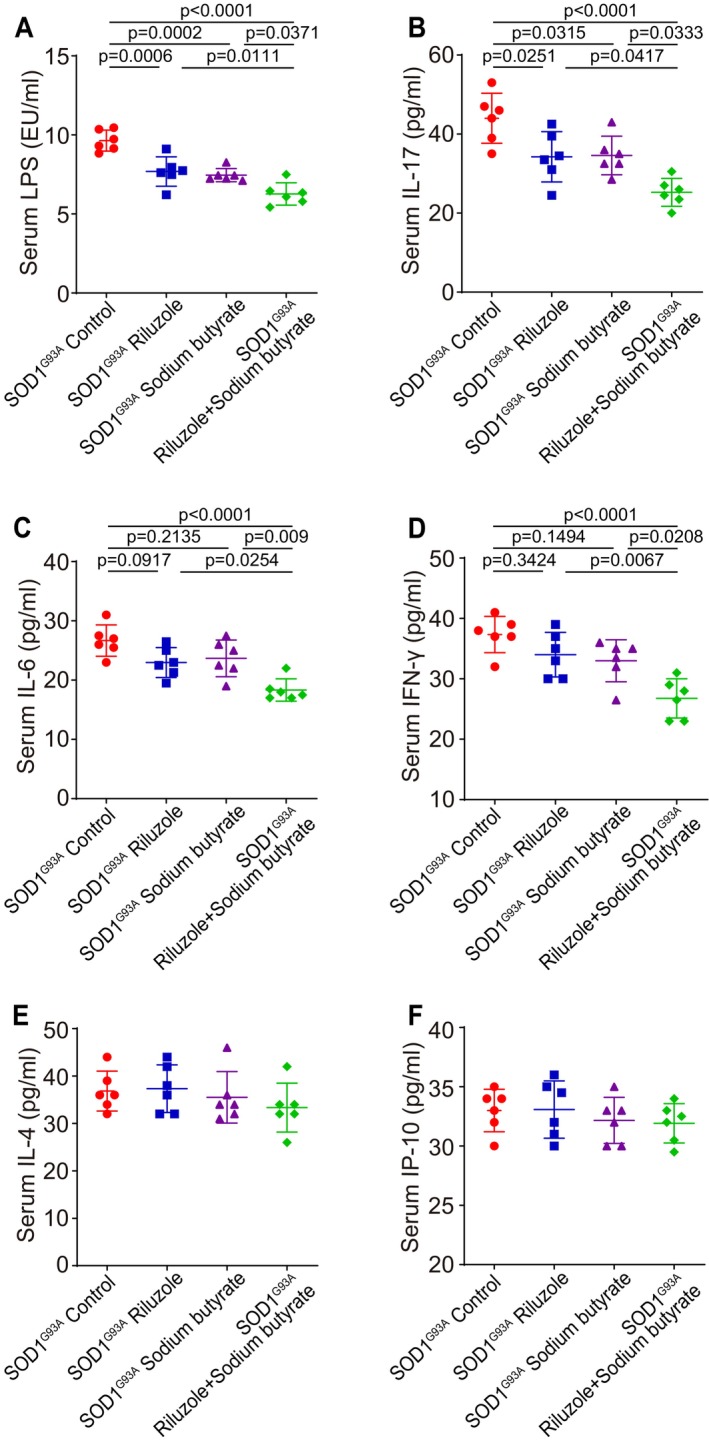
Reduced serum LPS, IL‐17, IL‐6, and IFN‐γ in Riluzole/sodium butyrate‐treated SOD1^G93A^ mice. The expression of serum inflammatory cytokines (A) LPS and (B) IL‐17 was decreased in all Riluzole‐only, sodium butyrate‐only, or Riluzole and sodium butyrate combination‐treated SOD1^G93A^ mice groups compared to control mice. Meanwhile, Riluzole and sodium butyrate combination‐treated SOD1^G93A^ mice had a significantly decreased serum LPS and IL‐17 expression compared to Riluzole or sodium butyrate‐only‐treated SOD1^G93A^ mice. Data are expressed as mean ± SD. *n* = 6, one‐way ANOVA test. The expression of serum inflammatory cytokines (C) IL‐6 and (D) IFN‐γ showed a slight decrease without statistical significance in Riluzole‐only and sodium butyrate‐only‐treated SOD1^G93A^ mice groups compared to SOD1^G93A^ mice without any treatment. But Riluzole and sodium butyrate combination‐treated SOD1^G93A^ mice had a significantly decreased serum IL‐6 and IFN‐γ expression compared to the no‐treatment SOD1^G93A^ mice. Meanwhile, Riluzole and sodium butyrate combination‐treated SOD1^G93A^ mice had a significant decrease in serum IL‐6 and IFN‐γ expression compared to Riluzole‐only or sodium butyrate‐only‐treated SOD1^G93A^ mice. Data are expressed as mean ± SD. *n* = 6, one‐way ANOVA test. The expression of serum inflammation cytokines (E) IL‐4 and (F) IP‐10 did not change in all groups. Data are expressed as mean ± SD. *n* = 6, one‐way ANOVA test. All *p*‐values are shown in the figures.

Intestinal leakage triggers bacterial LPS to blood. Thus, serum LPS could be tested by ELISA (Liu et al. 2010; Lu et al. 2010). As shown in Figure 5A, LPS were decreased in all mice with treatment. The expression of serum inflammatory cytokine IL‐17 was decreased in Riluzole, sodium butyrate, or Riluzole and sodium butyrate combination groups, compared to control mice (Figure 5B). Meanwhile, Riluzole and sodium butyrate combination in SOD1^G93A^ mice significantly decreased serum LPS and IL‐17 expression, compared to Riluzole or sodium butyrate‐only‐treated SOD1^G93A^ mice. The levels of serum inflammatory cytokines IL‐6 (Figure 5C) and IFN‐γ (Figure 5D) showed a slight decrease without statistical significance in Riluzole and sodium butyrate‐treated groups, compared to SOD1^G93A^ mice without any treatment. But mice with Riluzole and sodium butyrate combination had a significantly decreased serum IL‐6 and IFN‐γ, compared to the no‐treatment SOD1^G93A^ mice. However, serum cytokines IL‐4 and IP‐10 didn't change in all groups (Figure 5E,F), suggesting the role of Riluzole and sodium butyrate combination might be specific for certain inflammatory cytokines.

## 4 Discussion

In the current study, we investigate the combined roles of Riluzole and sodium butyrate for ALS therapy and explore new therapeutic targets for ALS by restoring barrier functions through the gut‐brain axis. Riluzole and sodium butyrate‐treated SOD1^G93A^ mice showed a significantly longer rotarod time, greater grip strength, and better barriers by TJs, compared with Riluzole or sodium butyrate‐only treatment. The aggregation of the h‐SOD1 mutated protein was tested as an indicator of ALS progression. More reduced SOD1^G93A^ aggregation was observed in the colon, spinal cord lumbar, and brain cortex with Riluzole and butyrate combination treatment compared with Riluzole or butyrate‐only treatment. The expression of TJ proteins (ZO‐1 and Claudin‐5) significantly increased in the colon, spinal cord lumbar, and brain cortex with Riluzole and butyrate combination treatment, compared with Riluzole‐only or sodium butyrate‐only treatment. An existing ALS drug and its synergistic effects with sodium butyrate provide insights into barrier function and ALS progression.

The mechanism of Riluzole in protecting the intestine in ALS is unknown (Kieran et al. 2004; Park et al. 2022). Our data not only showed the combined role of Riluzole and sodium butyrate in slowing down the disease progress through reserving barrier function and inhibiting inflammation, but also demonstrated that Riluzole‐only has beneficial impacts on the intestinal and blood–brain barriers. A previous study used I.P. treatment of Riluzole and sodium phenlebutyrate (NaPB) in the ALS mice (Del Signore et al. 2009). It showed that Riluzole/NaPB administration increased acetylation at H4 and increased NF‐κB p50 translocation to the nucleus in SOD1^G93A^ mice. However, the Riluzole and sodium butyrate has not been tested. In the current study, the combined role of Riluzole and sodium butyrate on the barrier functions and TJs was investigated through the gut‐brain axis. TJs control paracellular passage of substrates across the blood–brain barrier (BBB). Any damage to the TJ proteins may result in enhanced permeability. The combination of Riluzole and sodium butyrate not only improves gut barrier function as evidenced by decreased permeability and increased expression of TJ proteins, Claudin 5 and ZO‐1, in the intestine, but also enhances Claudin 5 and ZO‐1 in the spinal cord and brain. The reduced serum LPS and inflammatory cytokines also explain the influence of the Riluzole and sodium butyrate combination on restoring barrier damage and the function of spinal cord motor neurons and neuromuscular structures in ALS progress. Increased serum inflammatory cytokines (Joyce et al. 2011), LPS (Joyce et al. 2011), and aberrant microbiome have been reported in prior studies of human ALS (Zhang et al. 2009). Indeed, increased inflammatory cytokines IL‐17 and IL‐23 have been reported in the serum and cerebrospinal fluid of patients with ALS (Zhang et al. 2021). Previously, we have demonstrated increased serum IL‐17 in SOD1^G93A^ ALS mice, as compared to wild‐type mice (Rentzos et al. 2010). The combination of Riluzole and sodium butyrate had a significantly decreased serum LPS and IL‐17 and a slightly reduced IL‐6 and IFN‐γ in our current study. We believe that the Riluzole and sodium butyrate combination was able to maintain intestinal barrier functions through reducing serum LPS and inflammatory cytokines and enhancing TJ proteins.

Previous studies in inflammatory bowel disease have shown that butyrate may contribute to the restoration of the tight junction barrier by affecting the expression of Claudin‐2, Occludin, Cingulin, ZO‐1, and ZO‐2 [reviewed by Ploger et al. (2012)]. The mechanisms are through the inhibition of histone deacetylase and reduction of inflammatory cytokines (e.g., tumor necrosis factor α and interleukin 13). Butyrate is reported to protect the TJs, for example, through the cellular signaling pathways such as AMP‐activated protein kinase (AMPK) (Jan and Harm‐Jan 2013), Protein kinase B (also known as Akt) (Yan and Ajuwon 2017), and protein kinase C β (Miao et al. 2016). A recent study has shown that *n*‐butyrate upregulates Claudin‐23 expression through a transcriptional factor SP1 and the AMPK pathway in the intestine (Xu et al. 2023). The mechanism underlying the synergistic effects of Riluzole and sodium butyrate in regulating TJ expression remains unknown. We speculate that the key impacts are through the inhibition of histone deacetylase and reduction of inflammatory cytokines by both Riluzole and sodium butyrate. Moreover, the Riluzole and sodium butyrate combination had a significant impact on decreasing aggregated h‐SOD1^G93A^ in the intestine, spinal cord, and brain cortex, compared to Riluzole or sodium butyrate‐only‐treated SOD1^G93A^ mice. It will be a future research topic to better understand the molecular mechanisms of the synergistic effects of Riluzole and sodium butyrate on barrier function and disease progression of ALS through the gut‐neuron axis.

Emerging evidence suggests that altered microbiome contributes to the development or progression of ALS (Zhang et al. 2017, 2021; Martin et al. 2022; Rowin et al. 2017). ALS patients exhibited a noticeable change in their gut microbiome, primarily characterized by a significantly lower diversity of bacteria compared to healthy individuals (Rowin et al. 2017; Blacher et al. 2019). ALS patients with abnormalities in the gut microbiota might have a possible effect on disease severity, with shorter survival in ALS patients with a greater ratio of *Firmicutes* to *Bacteroides* (Lee et al. 2024). SOD1^G93A^ mice showed an abnormal microbiome profile with a reduced population of butyrate‐producing bacteria (Zhang et al. 2017). In SOD1^G93A^ mice fed with sodium butyrate, intestinal microbial homeostasis was restored, gut integrity was improved, and life span was prolonged, compared to those in mice without treatment (Zhang et al. 2017, 2021). Riluzole was reported to be significantly metabolized by 40 bacteria strains screened (Zimmermann et al. 2019; Boddy et al. 2021), affecting the gut microbiome through the metabolism and circulation (Gotkine et al. 2020). If the treatment with sodium butyrate‐only or Riluzole‐only can change gut microbiome profile and function, the combination of Riluzole and sodium butyrate likely impacts the gut microbiome. To investigate specific microbiome changes after the combined treatment can be our future research topic.

Taken together, our data suggest that the combination of Riluzole and sodium butyrate is more efficient and effective than Riluzole‐only or sodium butyrate‐only treatment in delaying ALS progress. Restoring barrier function and inhibiting chronic inflammation in the intestine, spinal cord, and brain provides a potential therapeutic strategy for ALS. Our study opens a new avenue in targeting the gut‐neuron axis for treating ALS.

## Author Contributions

Y.Z. performed the cellular and animal studies, the detailed analyses of the results; Y.Z. and K.R. performed animal studies; Y.Z. and J.S. prepared the Figures and the draft text; Y.X. contributed to the statistical analysis of data and the draft text; and J.S. obtained funds, designed the study, and directed the project. All authors contributed to the writing of the manuscript.

## Conflicts of Interest

The authors declare no conflicts of interest.

## Data Availability

All the data related to this paper are deposited in DOI: 10.5281/zenodo.15098540.
